# Effects of Bullying Forms on Adolescent Mental Health and Protective Factors: A Global Cross-Regional Research Based on 65 Countries

**DOI:** 10.3390/ijerph19042374

**Published:** 2022-02-18

**Authors:** Xiaoou Man, Jiatong Liu, Zengxin Xue

**Affiliations:** 1 School of Humanities and Law, Northeastern University, No. 195 Chuangxin Road, Hunnan District, Shenyang 110169, China; jiatong_liu@outlook.com; 2 College of Economics and Management, Zhejiang Agriculture and Forestry University, No. 666 Wusu Road, Lin’an District, Hangzhou 310000, China; zengxinxue@outlook.com

**Keywords:** adolescent, forms of bullying, parental support, mental health, global research

## Abstract

Adolescent bullying is a public health issue of great global concern. Given the serious negative effect of bullying on adolescent mental health, it is critical to seek protective factors to protect adolescent mental health. From a global cross-regional perspective, the study aims to explore the relationship between forms of bullying and adolescent mental health and the role of parental support as a protective factor. Data were drawn from adolescents aged 12–17 years in 65 countries from the Global School-based Student Health Survey between 2003 and 2015. After controlling the state-fixed effects, individual adolescent behavior, and family factors, the ordinary least squares model was used to analyze the influence of bullying frequency and forms of bullying on adolescent mental health. The results found that the prevalence of bullying in the sample of 167,286 adolescents was 32.03%, with the highest prevalence of bullying in the sample countries in Africa. Verbal bullying had the highest prevalence and the most significant negative effect on adolescent mental health. The study also discussed the differences in bullying among adolescents by gender, age, and region. “Parental supervision”, “parental connectedness” and “parental bonding” played a positive and protective role in the mental health of adolescents who experienced bullying.

## 1. Introduction

Bullying is intentional and repeated aggressive behavior toward another person in which there is a real or perceived power imbalance, and the victim of bullying feels vulnerable and powerless to protect themselves [1,2,3]. Bullying includes physical assault, verbal abuse, and neglect [4]. Globally, bullying is widespread among adolescents. In a 2018 report by UNICEF, more than one-third of students aged 13–15 worldwide said they had experienced different forms of bullying [5]; data published by the World Health Organization in 2020 showed that more than 100 million children worldwide died each year from violence, including severe domestic violence as well as bullying [6]. In a survey involving 40 developing countries, the results showed that an average of 42% of boys and 37% of girls had experienced or were experiencing bullying [7].

Evidence from several longitudinal studies on the effects of bullying suggests that experiencing bullying, especially in adolescence, can severely impair a person’s physical, psychological, and social functioning, leading to risky behaviors [8], anxiety [9], depression [3,10], lower levels of academic achievement [11,12], suicidal ideation, suicidal behavior, or self-harm [13,14,15]. At the same time, as a deliberate, repetitive act of aggression that occurs when there is a power imbalance between the perpetrator and the victim, the perpetrator repeats the bullying against the victim, and the repetition tilts the “balance” between the perpetrator and the victim, making it difficult for the victim to escape from the situation [2,4], thus having a lasting psychological effect on the victim [16,17]. This has a long-lasting effect on the victim’s psyche. Research has shown that the frequency of bullying is one of the factors that affect adolescent mental health. Adolescents are more likely to experience more severe depression when they are bullied more frequently [4], and some victims of bullying may even become perpetrators of bullying, harming peers or others [18,19,20].

In recent years, some studies have also begun to further explore the effects of different forms of bullying on adolescent mental health, and found that the form of bullying is also an essential factor affecting adolescent mental health. The first was to explore what forms of bullying had a profounder effect on adolescent mental health, but most of the current studies by researchers on this issue have been conducted in individual countries or regions and have not reached uniform conclusions, e.g., Maunder et al. (2010) conducted a survey of students, teachers, and staff in four secondary schools in England, and a total of 1302 people participated in this survey, and the results found that physical bullying was the most harmful to students [21]; Chen et al. (2012) selected a middle school in Taiwan, China, and conducted two samples (605 students and 869 students) and found that relational bullying such as rumor spreading and cyberbullying were more harmful than physical and verbal bullying [22]; Thomas et al. (2016) selected 10,273 secondary school students in the first adolescent health survey conducted in 2009 in Victoria, Australia, and found that neglect had the strongest association with mental health among four forms of bullying (teased or called names, spread rumors, neglect and physical bullying) [4]. In a representative cross-sectional standardized survey conducted by Baier et al. (2018) in a federal state of Germany (10,638 students in the 9th grade), psychological cyberbullying was found to be the most important influence on the mental health of boys and girls, followed by relational bullying from peers or from teachers, and girls’ mental health was associated with sexual cyberbullying. There was no significant effect between physical bullying and mental health [23].

The second was to focus on the effect of different forms of bullying on adolescent mental health under the gender group. For example, Turner et al. (2013) selected 1874 students from middle and high schools in North Carolina to explain the results of the effects of different forms of bullying (physical, verbal and cyber) on mental health (including depression and suicidal intention) and found that females had higher levels of depression after cyberbullying compared with males, and there was no significant difference in suicidal intent after either form of bullying for either males or females [24]. Shongwei et al. (2021) used the database of the 2013 Eswatini Global School-based Student Health Survey to examine gender differences in the effects of different forms of bullying on mental health in a sample of 2920 children aged 15–17 years, and found that both boys and girls felt lonely and feared re-victimization after being bullied [25]. Using data from U.S. Youth Risk Behavior Surveillance System in 2015, Kim et al. (2019) found that school bullying had a greater negative psychological effect on girls than on boys [26]. Wang et al. (2009) selected a sample of 7182 U.S. adolescents in grades 6 to 10 based on the 2005 Health Behavior in School-Aged Children Survey, and found that boys were more likely to involve physical or verbal bullying and girls were more likely to be involved in relational bullying [27]. Very few studies have focused on the effects of different forms of bullying on adolescent mental health according to age groups, and Yen et al. (2014) found that adolescents in middle school had more severe mental health problems after bullying than those in high school [28].

In addition to exploring the negative effects of bullying on adolescents, there were very few studies that analyze the role and effect of protective factors in preventing the occurrence of multiple forms of violence as positive actions to build resilience in children, in terms of protective factors [29,30,31]. For example, Biswas et al. (2020) used data from the Global School-based Student Health Survey to divide protective factors into parental support and peer support, and explored the effect of each on the mental health of adolescents following bullying [32].

Although some studies have been conducted on the effect of bullying on adolescent mental health, there are still the following research gaps: Firstly, for the global prevalence of adolescent bullying, the current studies are mostly limited to one country or a few regions [33,34], the findings are not consistent across countries, and there is a lack of cross-regional comparative studies. Secondly, in addition to focusing on the effect of bullying on the mental health of adolescents as a whole and different gender groups, there are not enough studies on the effect of bullying on the mental health of different adolescent subgroups. Adolescents are at a critical stage of development and the influence of age on their behaviors is crucial, but there is a lack of research discussing the effect of different forms of bullying on mental health according to age groups. Thirdly, current research has focused more on the risk factors of adolescent bullying and not enough on protective factors [31,35,36]. Therefore, to address these limitations, this study attempts to analyze the frequency of bullying, the prevalence of different forms of bullying, and the effects of both on adolescent mental health in 65 countries from a cross-regional comparative perspective, and to explore the differences in the effects of different forms of bullying on adolescent mental health by gender and age groups in different regions. In addition, the study focused on parental support as a protective factor to examine the relationship between parental support and the mental health of adolescents who experienced bullying, and the mental health of adolescents who experienced different forms of bullying. The following were our hypotheses:

**Hypothesis 1** **(H1).** *Forms of bullying would be associated with adolescents’ psychological well-being.*

**Hypothesis 2** **(H2).** *Forms of bullying would have significantly different effects on different genders and ages.*

**Hypothesis 3** **(H3).** *Parental support would play a moderating role in psychological well-being of adolescents who experienced bullying.*

## 2. Materials and Methods

### 2.1. Data and Sample

Global School-based Student Health Survey (GSHS) is a World Health Organization international survey of adolescents that uses primarily standardized, self-administered questionnaires to make results comparable between countries. The core questionnaire looks at 10 domains of key factors affecting adolescent health, including tobacco use, alcohol abuse, drug use, diet, hygiene, physical activity, sexual behavior, violent behavior, and unintentional injuries, protective factors, and mental health. For the actual survey, the GSHS was approved by national governments and sponsored or organized by official agencies at the national level (usually by Ministry of Health or Education, and an institutional review board or ethical committee), using a school-class two-stage whole-group sampling method. Questionnaires were translated into the national language for student comprehension. After excluding the samples with missing data, countries covering the key variables of this study were selected, using the most recent data available for each country, and the final sample was drawn from survey data from 2003 to 2015, for a total of 167,286 samples from 65 countries, 5 regions (21,501 samples from Africa, 59,326 samples from Americas, 23,222 samples from Eastern Mediterranean, 13,301 samples from South East Asia, 49,936 samples from Western Pacific).

### 2.2. Ethics Statement

GSHS received ethics approval from each country. Written informed consent was obtained from participants or guardians prior to the survey, and privacy protections were obtained. The current study used publicly available data.

### 2.3. Measures

#### 2.3.1. Dependent Variable: Mental Health

“Mental health”: Mental health was measured based on the two indicators of loneliness and anxiety in the questionnaire [37], with the questions “During the past 12 months, how often have you felt lonely / been so worried about something that you could not sleep at night?”. In order to visually explain the effect of bullying on adolescent mental health, this paper recoded the responses to the above measurement questions as “1 = always, 2 = most of the time, 3 = sometimes, 4 = rarely, 5 = never”. The current methods for comprehensive index measurement include subjective weighting method and objective weighting method. The subjective weighting method determines the weight based on the researcher’s subjective attention to the evaluation indicators, and the objective weighting method is based on the correlation between the indicators or indicators [38]. The degree of dispersion of information determines the weight. In order to eliminate the subjective arbitrariness of determining weights, following Huang’s research [39], this study chose the entropy method in the objective weighting method to determine the weights between the various indicators of the observed variable “mental health”, and used the information carried by the entropy value to calculate the “mental health”. According to the entropy method, the study determined the weight of each index as follows: there were N samples and M evaluation indexes, which were the value of the j-th index of the i-th sample.

Since the various indicators of sample mental health had different dimensions and orders of magnitude, the range standardized formula was used to process the indicators, and the absolute values of the indicators were converted into relative values to solve the homogeneity problem of various indicators, as shown below:

$$Z_{\mathrm{ij}}=\frac{x_{\mathrm{ij}}-{\mathrm{minx}}_{j}}{{\mathrm{maxx}}_{j}-{\mathrm{minx}}_{j}}$$

The formula following shows the standardized value of the j-th index of the i-th sample, the minimum and maximum value of the j-th index. In order to ensure the non-negativity of the calculation results, this study would shift the coordinates by 1 unit:

$$x_{\mathrm{ij}}^{\prime }=Z_{\mathrm{ij}}+1$$

Finally, the standardized values were used to calculate the mental health “MH” of adolescents in the sample:

$$\mathrm{MH}=\sum_{i=1}^{m}w_{j}P_{\mathrm{ij}}=\Sigma [\frac{1-e_{j}}{\sum_{i=j}^{m}\left(1-e_{j}\right)}\times \frac{x_{\mathrm{ij}}^{\prime }}{\sum_{i=j}^{n}x_{\mathrm{ij}}^{\prime }}]$$

The formula above represents the entropy value of the j-th index, k = 1/In(n); n is the sample size, which is the weight of the j-th index of the i-th sample. Finally, the evaluation score of “mental health” was calculated as 1.02–9.89, and the higher the score, the better the mental health status.

#### 2.3.2. Independent Variables: Frequency and Forms of Being Bullied

“Frequency of being bullied”: “Frequency of being bullied” was measured by the question “During the past 30 days, on how many days were you bullied?” and recoded (1 = 1 to 5 days, 2 = 6 to 19 days, 3 = more than 20 days). The larger value represented the higher frequency of being bullied. 

“Forms of being bullied”: “Forms of being bullied” was measured by the question “During the past 30 days, how were you bullied most often?” and recoded (1 = physical bullying, 2 = verbal bullying, 3 = neglect).

#### 2.3.3. Control Variables

Previous studies have reported that individual factors contributing to adolescent mental health, such as age, gender [40], substance use [26,41], weight status [42] and family socioeconomic status [43]. Therefore, the study used the following variables related to mental health of adolescents in GSHS as control variables, including age, gender, physical well-being, cigarette smoking, alcohol use, proxy of family socioeconomic status, number of close friends and frequency of missing school.

“Weight status” was measured by the value of body mass index (BMI), calculated with two adolescents’ indicators of height and weight, and recoded (1 = underweight, 2 = normal weight, 3 = overweight) [44,45]. “Cigarette smoking” and “alcohol use” were measured by the questions “During the past 30 days, on how many days did you smoke cigarettes / have at least drink containing alcohol?” and recoded (1 = less than 5 days, 2 = 6–19 days, 3= more than 20 days).

According to a prior study [14], “proxy of family socioeconomic status” was measured by the question “During the past 30 days, how often did you go hungry because there was not enough food in your home?” and recoded (1 represents “low”, 5 represents “high”). The larger value represented the higher family socioeconomic status.

“Number of close friends” was measured by the question “How many close friends do you have?” (0 = 0 friends, 1 = 1 friend, 2 = 2 friends, 3 = 3 or more friends). “Frequency of missing school” was measured by the question “During the past 30 days, on how many days did you miss classes or school without permission?” (1 = less than 2 days, 2 = 3–9 days, 3 = more than 10 days). 

#### 2.3.4. Protective Factors: Parental Supports

Protective factors were assessed by parental supports. As critical factors of resiliency, parental supports included parental supervision, parental connectedness and parental bonding [34,35], based on the questions “how often did your parents or guardians check to see if your homework was done?”, “how often did your parents or guardians understand your problems and worries?”, and “how often did your parents or guardians really know what you were doing with your free time?”, and assessed by frequency in the past 30 days (1 represents “never”, 5 represents “always”).

### 2.4. Statistical Analysis

Firstly, the study conducted descriptive statistics on the overall prevalence of maltreatment and the prevalence of different forms of maltreatment among adolescents aged 12–17 years in 65 sample countries among five regions, and to visualize the differences in the distribution of bullying across regions, a global distribution of bullying rates among adolescents in the 65 sample countries was drawn. Secondly, an ordinary least squares model was used to analyze the effects of bullying frequency and different forms of bullying on adolescent mental health. In the model estimation, state-fixed effects were controlled for in addition to the effects of the above-mentioned control variables on adolescent mental health. The study further regressed subgroups by gender and age to estimate differences in the effects of bullying exposure, bullying frequency, and forms of bullying on adolescent mental health by gender and by age (under 15, over 15) across continents, respectively. The reason for choosing 15 years as the age group cut-off was that in most countries, adolescents under 15 years are at the middle school level and those over 15 years are at the high school level, where they show more significant differences in psychological and behavioral aspects [28]. Finally, the study conducted moderation test to explore the protective factors of adolescent bullying, i.e., whether parental support could play a significant positive role in the effect of bullying on adolescent mental health. The study used Stata 15.0 to analyze the data and ArcGIS software for mapping.

## 3. Results

### 3.1. Sample Description

The descriptive statistics of the sample are shown in Table 1. The mean age of the sample adolescents was 14.14 years (SD = 1.20), of which 46.74% were male (78,187) and 53.26% were female (89,099). In terms of bullying prevalence, 32.03% of the 167,286 overall samples of adolescents aged 12–17 years had experienced bullying in the past 30 days of the survey. Regarding the frequency of bullying, 24.68% of adolescents were bullied for less than five days, less than 10% of adolescents were bullied for more than five days.

In terms of mental health, the mean of mental health of adolescents in the sample countries was 5.79 (SD = 1.82), which was in the middle to upper level. Among different regions, the mental health level of adolescents in South East Asia was the highest (M = 5.97, SD = 1.78), and African adolescents’ mental health level was the lowest (M = 5.47, SD = 1.92).

In terms of parental support, the mean values of “parental supervision”, “parental connectedness”, and “parental bonding” for the overall sample of adolescents were 2.94 (SD = 1.49), 3.00 (SD = 1.46), and 3.19 (SD = 1.44), respectively. The mean values of “parental supervision” ranged from “rarely” to “sometimes”, and the mean values of “parental connectedness” and “parental bonding” ranged from “sometimes” to “most of the time”.

### 3.2. Prevalence and Forms of Bullying across the Regions

Table 1 shows the prevalence of different forms of bullying in the six regions. Overall, verbal bullying had the highest prevalence (66.36%), followed by physical bullying (24.02%), and the neglect had the lowest prevalence (9.62%). Across regions, physical bullying was highest in Africa (28.98%) and lowest in the Americas (18.84%); verbal bullying was the opposite of physical bullying, highest in the Americas (71.09%) and lowest in Africa (61.75%); neglect was highest in South East Asia (11.10%) and lowest in Eastern Mediterranean (7.11%).

**Figure 1:**
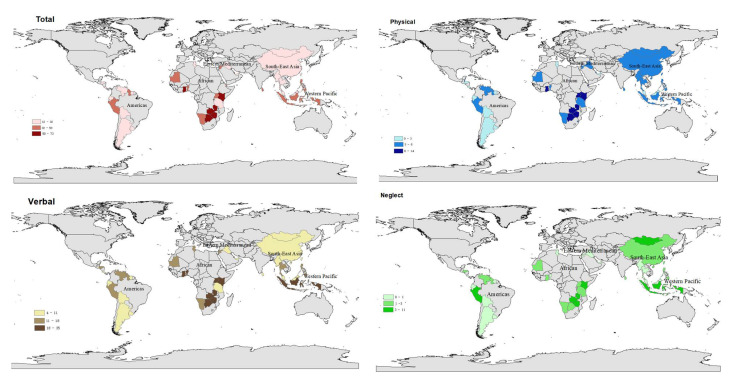
Prevalence of frequency of being bullied and forms of bullying in 65 countries.

Table 2 and Figure 1 show specifically the prevalence of bullying and different forms of bullying in each sample country. In terms of bullying prevalence, the African region had the highest prevalence of adolescent bullying at 47.36%, followed by Eastern Mediterranean (41.53%), South East Asia (33.19%), Western Pacific (27.58%), and the Americas (26.23%). In terms of sample countries, 5 of the 12 sample countries in Africa had more than half of the bullying prevalence, namely, Botswana (52.20%), Ghana (56.72%), Kenya (54.35%), Zambia (61.58%), and Zimbabwe (59.15%). In Americas, the prevalence of bullying was ranging from 47.14% in Peru to 12.50% in Barbados. The Eastern Mediterranean region had the highest bullying rate in the Occupied Palestinian Territory with over half (52.27%) and the lowest bullying rate in Iraq (27.45%). South East Asia also had more than half of adolescents in Indonesia (50.14%) as its highest bullying rate, and the lowest adolescent bullying rate was in Myanmar (19.51%). Samoa, in the Western Pacific region, had the highest prevalence of bullying among all countries in the sample, at 72.40%, while Malaysia (16.99%) had the lowest prevalence of bullying among adolescents in the Western Pacific region.

### 3.3. Effects of Bullying on Psychological Well-Being of Adolescents

After controlling for state-fixed effects, the study used OLS models to examine the effect of bullying and different forms of bullying on adolescent mental health, and the results are shown in Table 3. In Model 1, with bullying frequency as the core explanatory variable, the regression results showed that bullying frequency negatively affected adolescent mental health, with the largest negative effect on mental health for adolescents who had been bullied for more than 20 days in the past 30 days, with a 7.53 decrease in mental health (*p* < 0.001, CI: −7.72, −7.33). Model 2 further estimated the effects of different forms of bullying on adolescent mental health, and the results showed that verbal bullying negatively affected adolescent mental health mostly, with a 9.64 decrease in mental health (*p* < 0.001, CI: −9.89, −1.01). Physical and neglect also negatively affected adolescent mental health, with a 7.49 (*p* < 0.001, CI: −7.89, −7.10) and a 1.21 (*p* < 0.001, CI: −1.27, −1.15) decrease in mental health, respectively, which verified H1.

### 3.4. Effects of Bullying on Psychological Well-Being in Adolescents of Different Gender

Table 4 demonstrates the effects of bullying frequency and bullying form on the mental health of adolescents by gender. Overall, both bullying frequency and bullying form had a significant negative effect on both male and female adolescents in the sample across continents (*p* < 0.001). In the total sample, the negative effect of bullying frequency on the mental health of female adolescents was more significant than that of males (*p* < 0.001). Specifically, the negative effect of bullying frequency on the mental health of female adolescents was greater than that of males in the sample countries of the Eastern Mediterranean region, the South East Asian region, and the Western Pacific region; the negative effect of bullying frequency on males was greater when the bullying frequency was less than 19 days in the sample countries of the American region (*p* < 0.001).

Looking at the different forms of bullying, verbal bullying and neglect had a greater negative effect on overall female adolescents than on males, while physical bullying had a greater negative effect on overall male adolescents, supporting partial of H2. Across continents, all three forms of bullying had a significant negative effect on the mental health of male adolescents in Africa compared with females (*p* < 0.001); in the Americas, physical bullying had a greater negative effect on the mental health of male adolescents than females (*p* < 0.001), neglect had a greater negative effect on the mental health of female adolescents than males, and verbal bullying did not differ between the two sexes; in the Eastern Mediterranean region, physical bullying and verbal bullying had a greater negative effect on females than males (*p* < 0.001), and neglect had a more severe negative effect on males; in South East Asia, both physical bullying and verbal bullying had a more severe negative effect on females (*p* < 0.001), and neglect had a more severe negative effect on males (*p* < 0.001); in the Western Pacific, physical bullying and neglect had a more severe negative effect on female mental health, and verbal bullying had a more severe negative effect on male mental health.

### 3.5. Effects of Bullying on Psychological Well-Being in Adolescents of Different Ages

Table 5 demonstrates the effects of bullying and different forms of bullying on the mental health of adolescents of different ages across continents and their variability. In terms of bullying frequency, bullying frequency had a greater negative effect on the overall mental health of adolescents under the age of 15 than adolescents over the age of 15 (*p* < 0.001). In terms of forms of bullying, physical bullying, verbal bullying and neglect had a greater negative effect on the mental health of adolescents under 15 years old than adolescents over 15 years old in the total sample (*p* < 0.001) as hypothesized. Among the regions, the negative effect of neglect on the mental health of adolescents over the age of 15 was more significant in the sample countries of the Western Pacific region (*p* < 0.001), and the negative effect of physical bullying and verbal bullying on the mental health of adolescents under the age of 15 was more significant (*p* < 0.001); the situation in the other regions was consistent with that of the overall sample.

### 3.6. The Protective Effect of Parental Support on the Psychological Well-Being

To test the potential moderating role of parental support as a protective factor on adolescent mental health after bullying, the study conducted the procedure to test significant interactions. The results from Table 6 show that being bullied was negatively associated with mental health in three models (*p* < 0.001). Significant interaction effects between parental supervision and being bullied (*p* < 0.001), between parental connectedness and being bullied (*p* < 0.001), between parental bonding and being bullied (*p* < 0.001) were found to be positively associated with psychological well-being, indicating that the moderating effect of parental support occurred in the protection of mental health of adolescents who experienced being bullied as H3 hypothesized.

Table 7 shows the results of the effect of parental support on the mental health of adolescents following different forms of bullying. Among them, “parental connectedness” had a positive protective effect on the mental health of adolescents after verbal bullying or peer neglect, i.e., the more parents understand the adolescents’ distress after verbal bullying or neglect at school, the higher the level of mental health of the adolescents, and the frequency of parental understanding increases by one unit, the level of mental health increased by 8.71 units (*p* < 0.001) and 1.05 units (*p* < 0.001), respectively; “parental bonding” had a positive restorative effect on the psychological health of adolescents who were verbally bullied, i.e., for each unit increase in the frequency of “parental bonding”, the psychological health level of adolescents who were verbally bullied increased by 2.47 units (*p* < 0.05).

## 4. Discussion

The study examined the overall prevalence of bullying among adolescents and the prevalence of different forms of bullying in a total of 167,286 sample in five regions, and further analyzed the effect of different forms of bullying on adolescent mental health, the protective role of parental support, and the main findings were as follows:

Firstly, adolescent bullying cannot be ignored, with the highest prevalence of verbal bullying. Our study showed that the overall prevalence of bullying among adolescents in the 167,286 sample countries was 32.03%, a result that was consistent with the previous UNICEF report published in 2018 that more than one-third of students aged 13–15 worldwide experienced bullying. The results of Biswas et al. (2020) and Elgar et al. (2015) cross-regional comparative studies on bullying and violence among adolescents were generally consistent with the results of the two studies on the prevalence of bullying among adolescents, which were 31% [32] and 30% [8], respectively. From the results of the cross-regional comparison, the highest prevalence of bullying among adolescents (47.36%) was found in the sample countries in the African region, which may be related to the low-income level, poorer schools, and social environment, war, and riots in the African region [46]. In terms of the prevalence of different forms of bullying, verbal bullying had the highest prevalence (66.36%), followed by physical bullying (24.02%), and neglect had the lowest prevalence (9.62%). The results of a survey conducted by Scheithauer et al. (2006) in Germany with students in grades 5–10 [47], and the results of the prevalence of six forms of bullying among 2667 Italian secondary school students, obtained by Vieno et al. in 2011 using the results of the Health Behavior in School-aged Children Survey database, also both showed the highest prevalence of verbal bullying, consistent with the findings of this paper [48]. This suggested that verbal bullying, which takes the form of making fun of a peer’s race, nationality, color, creed, body, and appearance, was the most prevalent and most likely to occur among adolescents because it was the most recognizable and less costly to occur. However, it was worth pointing out that the findings for the prevalence of physical bullying and neglect in this study differ slightly from those of the two studies mentioned above, due to the different criteria used to measure them.

Secondly, compared with physical bullying and neglect, verbal bullying had the most serious negative effect on adolescent mental health. Not only did verbal bullying had the highest prevalence of the three forms of bullying, but it also had the most serious negative effect on adolescent mental health for two main reasons: firstly, verbal bullying occurred most frequently, and according to the study, the frequency of bullying significantly and negatively affects adolescent mental health, so the lower the level of mental health when adolescents suffered frequent ridicule or name-calling from peers; secondly, from the perspective of social identity theory, this highly discriminatory ridicule led to negative mental health outcomes, especially for adolescents with extremely strong identity, and this discrimination increased their psychological distress [49].

Thirdly, overall, the frequency of bullying had a greater negative effect on the mental health of female adolescents compared with male adolescents, which was consistent with the findings of a recent study conducted in the United States that school bullying had a greater effect on psychological depression in females than in males [26]. In addition, physical bullying had a greater negative effect on the mental health of male adolescents, and verbal bullying and neglect had a greater negative effect on the mental health of female adolescents. This was generally consistent with previous research finding that depressive symptoms were more pronounced after active forms of bullying (i.e., physical bullying) in boys and after passive forms of bullying (i.e., verbal and relational bullying) in girls [28,50]. This would require further exploration of the effect of different forms of bullying on the mental health of male and female adolescents in specific regions. While we need to protect boys and girls equally from bullying, countries also need to consider the gender differences in the occurrence and effect of different forms of bullying in their countries and pay targeted attention to adolescents who are victims of bullying.

Fourth, the frequency of bullying had a more significant negative effect on the mental health of adolescents under the age of 15, and different forms of bullying also had a more significant negative effect on the mental health of adolescents under the age of 15. Previous studies have found that the odds of bullying are higher for younger adolescents (under 15) [25,51]. Compared with younger adolescents, older adolescents (15 years and older) were more aware of self-concept and self-regulation in terms of self-perception and psychological construction [49], so both the frequency of bullying and the different forms of bullying had a more significant negative effect on the mental health of adolescents under the age of 15. In addition, the study showed regional differences in mental health of adolescents in different age groups after various forms of bullying, which provided a basis for the development and implementation of intervention policies in each region or country.

Finally, in terms of protective factors, “parental supervision”, “parental connectedness” and “parental bonding” played positive roles in the relationship between bullying and adolescent mental health. Positive relationships, especially positive family relationships that provided intimacy, support, trust, emotional comfort, and a sense of belonging, are one of the key elements of resiliency [52]. In such a family environment, even if adolescents were abused and bullied, they could still buffer the stress and shock from other aspects by increasing their self-efficacy, self-worth, and emotional belongingness [53]. “Parental connectedness” and “parental bonding” were important indicators of parent–child intimacy and emotional comfort, and played a positive role in adolescents’ resilience. However, there were no consistent conclusions to the role of “parental supervision”. Some studies have not found a significant link between parental supervision and mental health after bullying [54,55]. Others have identified the lack of parental supervision as a risk factor to adolescents’ mental health development [56], which is consistent with the current study. Future research would explore how the degree or the forms of parental supervision influence mental health when adolescents experience bullying.

Limited by the consistency of the GSHS database, this study suffered from the following shortcomings: Firstly, the countries or regions selected represent only some of the five regions. We did not contain the European continent because only one country provided useful data. Future studies would include more specific countries to explore the global adolescent bullying situation. Secondly, the GSHS used a self-administered questionnaire, and although self-administration was an acceptable way to collect data on adolescent bullying victimization, there was a limitation of possible shared method variance. Finally, we observed significant regional differences in the prevalence of different forms of bullying, including gender differences and age differences, and future research would consider social context and cultural heterogeneity to explain regional differences better and provide more possibilities for countries to implement adolescent bullying intervention programs.

## 5. Conclusions

Despite these limitations, our study contributed to the exploration of adolescent bullying in the following ways: firstly, unlike previous studies limited to individual countries or regions, our analysis covered 65 sample countries across five continents, providing more evidence for cross-regional comparative studies of adolescent bullying; secondly, in addition to focusing on bullying among adolescents as a whole and its effect on mental health, we focused on intergroup differences in adolescent subgroups (gender groups and age groups) to provide a basis for targeted development of specific intervention policies for different groups of adolescents. Finally, we focused on the potential protective factors of adolescent bullying and found that “parental supervision”, “parental connectedness” and “parental bonding” played a positive role in protecting the psychological health of adolescents who were bullied. The above findings suggested that, as a global public health problem, adolescent bullying should attract sufficient policy concern and practical intervention, and further establish a comprehensive adolescent social protection mechanism and protection system including family, school, and community.

## Figures and Tables

**Table 1:** Descriptive statistics of the sample (N = 167,286).

Variable	Percentage/Mean (SD)					
	Total	African	Americas	Eastern Mediterranean	South East Asia	Western Pacific
Independent variable						
Being bullied	32.03%	47.36%	26.23%	41.53%	33.19%	27.58%
Frequency of being bullied						
1–5 days	24.68%	35.68%	20.46%	31.31%	26.73%	21.34%
6–19 days	4.06%	6.60%	3.10%	5.52%	3.97%	3.44%
>20 days	3.28%	5.08%	2.67%	4.70%	2.50%	2.79%
Form of being bullied						
Physical	24.02%	28.98%	18.84%	24.71%	25.66%	24.26%
Verbal	66.36%	61.75%	71.09%	68.19%	63.24%	65.12%
Neglect	9.62%	9.26%	10.07%	7.11%	11.10%	10.61%
Dependent variable						
Psychological well-being	5.79 (1.82)	5.47 (1.92)	5.89 (1.81)	5.69 (2.00)	5.97 (1.78)	5.81 (1.70)
Control variable						
Age	14.14 (1.20)	14.45 (1.29)	13.89 (0.99)	13.85 (0.95)	14.05 (1.18)	14.45 (1.37)
Gender						
Male	46.74%	45.81%	46.77%	47.10%	45.20%	47.34%
Female	53.26%	54.19%	53.23%	52.90%	54.80%	52.66%
Weight status						
Underweight	26.63%	20.66%	17.29%	27.45%	48.43%	34.12%
Normal weight	63.69%	75.31%	73.17%	62.76%	47.86%	52.09%
Overweight	9.67%	4.03%	9.55%	9.80%	3.71%	13.79%
Cigarettes smoking						
<5 days	96.11%	97.72%	95.98%	95.96%	97.58%	95.24%
6–19 days	1.92%	1.13%	2.22%	2.01%	1.17%	2.05%
>20 days	1.98%	1.14%	1.80%	2.03%	1.26%	2.71%
Alcohol use						
<5 days	95.97%	96.73%	91.84%	99.58%	98.65%	98.17%
6–19 days	2.91%	2.08%	6.11%	0.31%	0.90%	1.19%
>20 days	1.12%	1.19%	2.05%	0.12%	0.44%	0.63%
Socioeconomic status	4.20 (1.01)	3.89 (1.09)	4.39 (0.93)	4.36 (1.03)	4.18 (1.05)	4.04 (1.01)
Close friendship	2.41 (0.92)	1.91 (1.04)	2.45 (0.91)	2.39 (0.91)	2.53 (0.83)	2.56 (0.84)
Frequency of missing school						
<2 days	91.58%	90.04%	90.93%	90.64%	94.77%	92.52%
3–9 days	6.62%	7.82%	6.89%	7.48%	4.04%	6.13%
>10 days	1.80%	2.14%	2.18%	1.87%	1.20%	1.35%
Parental supervision	2.94 (1.49)	3.14 (1.51)	2.97 (1.51)	3.37 (1.56)	3.15 (1.42)	2.57 (1.36)
Parental connectedness	3.00 (1.46)	3.11 (1.44)	3.16 (1.48)	3.16 (1.48)	2.98 (1.44)	2.81 (1.37)
Parental bonding	3.19 (1.44)	3.06 (1.43)	3.34 (1.46)	3.34 (1.50)	3.35 (1.39)	3.17 (1.36)

**Table 2:** Prevalence and forms of bullying across regions (N = 167,286).

Region	Being Bullied (%)	Physical (%)	Verbal (%)	Neglect (%)	Total Sample (n)
**Total**	**32.03**	**4.50**	**12.43**	**1.80**	**167,286**
**African**	**47.36**	**9.65**	**20.56**	**3.08**	**21501**
Benin	42.46	6.28	19.96	0.75	1067
Botswana	52.20	11.64	23.90	3.53	1134
Ghana	56.72	12.79	20.15	2.31	2119
Kenya	54.35	13.56	26.94	5.47	2138
Mauritania	47.46	6.30	15.50	2.10	1142
Namibia	45.59	8.27	17.19	1.80	2937
Seychelles	47.26	3.56	17.46	1.37	1094
Swaziland	31.28	5.91	11.66	0.92	2487
Uganda	43.09	11.66	20.52	2.54	1613
Tanzania	25.74	6.78	9.48	2.13	1593
Zambia	61.58	13.07	34.52	4.93	872
Zimbabwe	59.15	12.22	29.89	6.54	3305
**Americas**	**26.23**	**2.77**	**10.44**	**1.48**	**59326**
Antigua and Barbuda	26.87	3.55	12.75	0.55	1098
Argentina	25.07	1.79	9.97	1.32	19559
Bahamas	21.74	2.42	10.03	1.09	1196
Barbados	12.50	2.11	4.94	0.36	1376
Belize	30.29	4.05	12.07	1.40	1433
Bolivia	30.51	3.07	10.08	1.63	2511
British Virgin Is.	18.06	1.80	7.91	0.63	1113
Cayman Is.	26.51	5.26	11.50	1.36	1026
Costa Rica	19.34	1.34	8.68	1.39	2166
Curacao	26.59	0.73	12.90	1.17	2046
El Salvador	25.30	3.43	8.85	1.99	3965
Ecuador	22.50	1.39	8.76	1.46	1507
Grenada	27.77	3.76	12.65	1.28	1091
Guyana	38.32	4.78	13.56	2.19	1777
Honduras	31.00	1.89	12.15	2.69	1374
Jamaica	36.91	5.37	14.36	1.46	1024
Montserrat	28.77	5.48	12.33	10.96	146
Peru	47.14	4.24	17.83	4.51	2238
St. Kitts and Nevis	22.07	4.16	8.77	0.89	1346
St. Lucia	25.20	2.87	10.76	1.43	976
St. Vincent and the Grenadines	28.48	5.43	11.07	1.43	976
Suriname	26.26	0.91	7.55	0.30	994
Trinidad and Tobago	15.77	2.90	6.30	0.50	2207
Uruguay	18.98	0.74	9.60	1.26	2689
Venezuela	32.36	6.98	12.40	1.81	3483
**Eastern Mediterranean**	**41.53**	**5.42**	**14.95**	**1.56**	**23222**
Bahrain	40.06	10.92	20.17	1.26	714
Djibouti	40.06	10.92	20.17	1.26	714
Iraq	27.45	6.10	7.12	0.58	1377
Jordan	41.57	5.35	14.96	1.47	1364
Lebanon	23.79	5.08	8.44	0.33	1812
Occupied Palestinian territory	52.27	5.67	18.22	1.96	11614
Qatar	37.40	4.28	13.58	1.67	1377
Tunisia	30.28	3.79	11.69	1.58	2087
United Arab Emirates	22.10	2.64	8.41	1.20	2163
**South East Asia**	**33.19**	**4.68**	**11.53**	**2.02**	**13301**
Indonesia	50.14	4.49	20.20	4.53	2559
Maldives	32.73	3.17	9.84	1.58	1830
Myanmar	19.51	4.77	7.84	1.66	1927
Sri Lanka	36.00	4.26	6.96	2.47	2228
Thailand	28.23	6.42	12.94	0.74	2288
Timor Leste	28.72	4.66	9.48	0.81	2469
**Western Pacific**	**27.58**	**3.86**	**10.37**	**1.69**	**49936**
Brunei	20.40	1.45	7.41	1.32	2417
China	28.29	5.30	8.71	1.83	7780
Cook Is.	27.14	3.95	9.54	1.32	608
Kiribati	34.96	8.24	18.27	0.64	1396
Malaysia	16.99	1.51	7.88	0.63	23476
Mongolia	27.62	4.18	4.71	4.95	4885
Nauru	38.72	10.44	15.15	0.67	297
Philippines	48.22	4.29	15.46	3.70	3544
Samoa	72.40	13.06	34.66	2.91	1685
Solomon Is.	63.55	10.61	24.68	3.58	782
Tonga	51.00	8.54	20.67	2.00	1698
Tuvalu	29.62	6.87	7.04	0.65	611
Vanuatu	65.92	14.93	21.80	2.11	757

**Table 3:** Effects of bullying on psychological well-being of adolescents (N = 167,286).

	Model 1 (95% CI)	Model 2 (95% CI)
Frequency of being bullied		
1–5 days	−1.22 *** (−1.27, −1.18)	
6–19 days	−1.43 *** (−1.47, −1.38)	
>20 days	−7.53 *** (−7.72, −7.33)	
Form of being bullied		
Physical		−7.49 *** (−7.89, −7.10)
Verbal		−9.64 *** (−9.89, −1.01)
Neglect		−1.21 *** (−1.27, −1.15)
Age	−1.29 *** (−1.36, −1.23)	−1.27 *** (−1.33, −1.20)
Gender (Female)	−5.97 *** (−6.13, −5.81)	−5.80 *** (−5.96, −5.63)
Weight Status		
Normal weight	−1.28 *** (−1.46, −1.09)	−1.25 *** (−1.43, −1.06)
Overweight	−7.60 *** (−9.41, −3.42)	−4.47 *** (−7.48, −1.05)
Cigarettes smoking		
6–19 days	−3.43 *** (−4.02, −2.84)	−3.84 *** (−4.43, −3.25)
>20 days	−3.64 *** (−4.22, −3.05)	−4.16 *** (−4.75, −3.57)
Alcohol use		
6–19 days	−3.13 *** (−3.61, −2.65)	−3.23 *** (−3.71, −2.75)
>20 days	−3.84 *** (−4.61, −3.07)	−4.46 *** (−5.23, −3.69)
Socioeconomic status		
Middle-low	−2.64 *** (−3.32, −1.97)	−2.47 *** (−3.15, −1.80)
Middle	−2.64 (−8.25, 2.96)	2.07 (−3.55, 7.68)
Middle-high	2.66 *** (2.10, 3.22)	3.29 *** (2.73, 3.85)
High	6.15 *** (5.60, 6.69)	6.74 *** (6.20, 7.28)
Close friendship		
1 friend	1.27 *** (0.04, 1.71)	1.45 *** (1.05, 1.84)
2 friends	1.65 *** (1.27, 2.02)	1.89 *** (1.52, 2.27)
3 or more friends	3.58 *** (3.24, 3.91)	3.82 *** (3.48, 4.15)
Frequency of missing school		
3–9 days	−2.63 *** (−2.95, −2.30)	−2.85 *** (−3.17, −2.52)
>10 days	−4.08 *** (−4.69, −3.48)	−4.68 *** (−5.28, −4.07)
Parental supervision		
Rarely	5.98 *** (3.44, 8.53)	5.86 *** (3.31, 8.41)
Sometimes	1.25 (−2.37, 2.62)	5.66 (−1.93, 3.06)
Most of the time	6.48 *** (3.69, 9.27)	6.84 *** (4.04, 9.63)
Always	7.33 (4.79, 9.86)	6.97 *** (4.43, 9.51)
Parental connectedness		
Rarely	5.98 *** (3.44, 8.53)	6.34 *** (3.65, 9.03)
Sometimes	1.25 (−2.37, 2.62)	5.96 (3.44, 8.49)
Most of the time	6.48 *** (3.69, 9.27)	1.92 *** (1.63, 2.20)
Always	7.33 *** (4.79, 9.86)	3.559 *** (3.32, 3.86)
Parental bonding		
Rarely	−9.73 *** (−1.26, −6.88)	−8.33 *** (−1.12, −5.47)
Sometimes	−7.61 *** (−1.03, −4.96)	−6.24 *** (−8.89, −3.59)
Most of the time	−1.99 (−4.85, 8.68)	1.45 (−2.72, 3.01)
Always	1.30 *** (1.03, 1.57)	1.48 *** (1.21, 1.75)
Constant	8.15 ***	7.96 ***
R^2^	0.17	0.17
N	167,286	167,286

Note: *** *p* < 0.001.

**Table 4:** Effects of bullying on psychological well-being in adolescents of different gender (N = 167,286).

	Total		African		Americas		Eastern Mediterranean		South East Asia		Western Pacific	
	Male	Female	Male	Female	Male	Female	Male	Female	Male	Female	Male	Female
Frequency of being bullied (X^2^)	293.43 ***		4.80		26.69 ***		284.47 ***		168.79 ***		67.34 ***	
1–5 days	−1.21 ***	−1.23 ***	−1.21 ***	−9.93 ***	−1.29 ***	−1.23 ***	−1.15 ***	−1.55 ***	−1.08 ***	−1.22 ***	−1.03 ***	−1.16 ***
6–19 days	−1.40 ***	−1.45 ***	−1.16 ***	−1.29 ***	−1.51 ***	−1.48 ***	−1.50 ***	−1.83 ***	−1.02 ***	−1.69 ***	−1.25 ***	−1.16 ***
>20 days	−7.01 ***	−7.94 ***	−6.74 ***	−7.10 ***	−7.54 ***	−8.25 ***	−6.15 ***	−8.46 ***	−6.44 ***	−8.50 ***	−5.95 ***	−7.36 ***
Form of being bullied (X^2^)	1.0 ***		73.69 ***		424.39 ***		547.14 ***		211.67 ***		224.02 ***	
Physical	−7.86 ***	−6.62 ***	−7.21 ***	−7.02 ***	−8.64 ***	−5.63 ***	−8.10 ***	−9.72 ***	−6.93 ***	−8.36 ***	−5.94 ***	−6.48 ***
Verbal	−9.55 ***	−1.01 ***	−9.37 ***	−9.17 ***	−1.07 ***	−1.07 ***	−1.20 ***	−8.13 ***	−1.11 ***	−8.62 ***	−8.39 ***	−8.05 ***
Neglect	−1.24 ***	−1.18 ***	−6.80 ***	−1.11 ***	−1.28 ***	−1.33 ***	−1.70 ***	−1.40 ***	−1.30 ***	−1.14 ***	−1.25 ***	−9.89 ***

Note: *** *p* < 0.001.

**Table 5:** Effects of bullying on psychological well-being in adolescents of different ages (N = 167,286).

	Total		African		Americas		Eastern Mediterranean		South East Asia		Western Pacific	
	≥15	<15	≥15	<15	≥15	<15	≥15	<15	≥15	<15	≥15	<15
Frequency of being bullied (X^2^)	218.44 ***		10.73 *		8.11 *		35.85 ***		19.10 ***		815.58 ***	
1–5 days	−1.07 ***	−1.25 ***	−9.92 ***	−1.20 ***	−1.16 ***	−1.31 ***	−1.23 ***	−1.40 ***	−7.83 ***	−1.32 ***	−1.01 ***	−1.13 ***
6–19 days	−1.30 ***	−1.46 ***	−1.09 ***	−1.36 ***	−1.46 ***	−1.50 ***	−1.68 ***	−1.64 ***	−1.08 ***	−1.41 ***	−1.00 ***	−1.31 ***
>20 days	−6.80 ***	−7.61 ***	−6.29 ***	−7.64 ***	−7.24 ***	−8.27 ***	−6.78 ***	−7.71 ***	−6.68 ***	−7.97 ***	−6.56 ***	−6.70 ***
Form of being bullied (X^2^)	211.83 ***		23.29 ***		25.00 ***		56.24 ***		70.41 ***		703.00 ***	
Physical	−5.97 ***	−6.72 ***	−7.14 ***	−7.20 ***	−6.21 ***	−7.82 ***	−8.80 ***	−8.86 ***	−5.56 ***	−7.60 ***	−5.65 ***	−6.23 ***
Verbal	−9.10 ***	−1.02 ***	−8.18 ***	−9.32 ***	−1.02 ***	−1.08 ***	−9.49 ***	−9.98 ***	−9.71 ***	−9.97 ***	−7.71 ***	−8.26 ***
Neglect	−1.09 ***	−1.32 ***	−8.04 ***	−8.65 ***	−1.19 ***	−1.31 ***	−1.40 ***	−1.56 ***	−1.18 ***	−1.25 ***	−9.75 ***	−1.15 ***

Note: * *p* < 0.05. *** *p* < 0.001.

**Table 6:** Tested moderation models with psychological well-being as outcomes predicted by being bullied, parental support and multiplicative interaction terms (N = 167,286).

	B	R^2^	95% CI
Model 1		0.15	
Being bullied	−9.98 ***		(−9.60, −1.03)
Parental supervision * Being bullied	3.22 ***		(2.13, 4.31)
Constant	8.41 ***		(8.29, 8.54)
Model 2		0.16	
Being bullied	−1.14 ***		(−1.18, −1.10)
Parental connectedness * Being bullied	8.19 ***		(7.05, 9.34)
Constant	8.43 ***		(8.30, 8.55)
Model 3		0.15	
Being bullied	−1.05 ***		(−1.09, −1.01)
Parental bonding * Being bullied	4.93 ***		(3.78, 6.07)
Constant	8.43 ***		(8.30, 8.55)

Note: *** *p* < 0.001.

**Table 7:** Association of protective effect of parental support with psychological well-being by forms of being bullied (N = 167,286).

	Physical	Verbal	Neglect
Parental supervision	−1.53	8.21	3.14
Parental connectedness	2.36	8.71 ***	1.05 ***
Parental bonding	2.23	2.47 *	8.98
Constant	0.00 ***	0.00 ***	0.00 **
N	7554	20,875	3031
R^2^	0.08	0.10	0.08

Note: * *p* < 0.05. ** *p* < 0.01. *** *p* < 0.001.

## Data Availability

The data are available online at https://www.cdc.gov/gshs/ (accessed on 10 December 2021).
